# Effects of Different Ions and Temperature on Corrosion Behavior of Pure Iron in Anoxic Simulated Groundwater

**DOI:** 10.3390/ma13122713

**Published:** 2020-06-15

**Authors:** Teng Li, Guokai Huang, Yanpeng Feng, Miao Yang, Lingyu Wang, Daqing Cui, Xian Zhang

**Affiliations:** 1 Department of Radiochemistry, China Institute of Atomic Energy, Beijing 102413, China; linyuwang2955@163.com (L.W.); daqing.cui@studsvik.se (D.C.); 2 School of Chemistry, Chemical Engineering and Life Sciences, Wuhan University of Technology, Wuhan 430070, China; guokaihuang@whut.edu.cn (G.H.); fengyanpeng1996@gmail.com (Y.F.); yangmiao@whut.edu.cn (M.Y.); 3 Department of Materials and Environmental Chemistry, Stockholm University, SE-106 91 Stockholm, Sweden; 4 The State Key Laboratory of Refractories and Metallurgy, Hubei Province Key Laboratory of Systems Science in Metallurgical Process, Collaborative Innovation Center for Advanced Steels, Wuhan University of Science and Technology, Wuhan 430081, China

**Keywords:** pure iron, groundwater, corrosion behavior, ions, temperature

## Abstract

As a typical material of the insert in high-level radioactive waste (HLW) geological disposal canisters, iron-based materials will directly contact with groundwater after the failure of a metallic canister, acting as a chemical barrier to prevent HLW leaking into groundwater. In this paper, anoxic groundwater was simulated by mixing 10 mM NaCl and 2 mM NaHCO_3_ purged by Ar gas (containing 0.3% CO_2_) with different added ions (Ca^2+^, CO_3_^2−^ and SiO_3_^2−^) and operation temperatures (25, 40 and 60 °C). An electrochemical measurement, immersion tests and surface characterization were carried out to study the corrosion behavior of pure iron in the simulated groundwater. The effects of Ca^2+^ on the corrosion behavior of iron is negligible, however, Cl^−^ plays an important role in accelerating the corrosion activity with the increased concentration and temperature. With increased concentrations of CO_3_^2−^ and SiO_3_^2−^, the corrosion resistance of iron is largely improved, which is attributed to the formation of a uniform passivation film. The independent effects of temperature on the corrosion behavior of iron are resulted from the repeated passivation–dissolution processes in the formation of the passivation film, resulting from the synergistic effects of CO_3_^2−^/SiO_3_^2−^ and Cl^−^. The formation of ferric silicate is dominant in the passivation film with the addition of SiO_3_^2−^, which effectively protects the iron surface from corrosion.

## 1. Introduction

The metallic canister is the first barrier to prevent high-level radioactive waste (HLW) from leakage in different countries. Although with the same concept on selecting the candidate materials for the canister, i.e., with good mechanical properties and corrosion resistance, the relevant countries have made relatively different choices. To be specific, a Cu canister with a nodular cast iron insert is adopted in Sweden and Finland [1,2]; a Cu coating on a welded steel vessel is being designed in Canada [3,4]; a stainless steel canister with a glass or ceramic waste form is being planned in the US [5]; and a stainless steel with cast iron is selected by France [6]. Iron-based materials are considered not only because of their mechanical performance but also their nature as reductants [1].

Pure iron has been proved to be able to reduce highly dissolved U(VI), Se(IV) and Tc(VII) to insoluble UO_2_, FeSe_2_ and TcO_2_ in an anoxic solution [1,7,8,9,10,11] since the airborne O_2_ in the deep geological repository will have been consumed completely by the iron minerals before the canister failure [12,13]. In case of canister failure, the intrusion of underground water together with a radiation effect may lead to the dissolution of toxic U(VI). However, based on the nature of pure iron, it can be predicted that the toxic dissolved U(VI) from HLW can be reduced to insoluble UO_2_, thereby not entering the biosphere. Considering this, the service behavior and development of corrosion products of iron-based materials in a groundwater environment may affect the reduction and inhibition of HLW. Therefore, it is of utmost significance to study the corrosion behavior of pure iron in a groundwater environment under anoxic conditions.

The lifetime of a canister is affected by environmental factors such as dissolved oxygen, composition of groundwater and temperature in the repository. Based on the surveys in different sites for repository [14], it demonstrates that the main cations are Na^+^, K^+^, Ca^2+^ and Mg^2+^, while the main anions are HCO_3_^−^/CO_3_^2−^, Cl^−^ and SO_4_^2−^. At the beginning period of the geological disposal, the temperature rises in a short period of time due to the release of residual heat from the high-level radioactive waste, and then decreases gradually. N.R. Smart and co-workers [15] reported that the corrosion rate of low-carbon steel in an anoxic simulated groundwater solution of Sweden at 30–85 °C is below 0.1 μm/y. C. Liu et al. [16] measured the average corrosion rate of low-carbon steel in aerobic and unsaturated bentonite after irradiation and thermal aging treatment at 90 °C. F. A. Martin et al. [17] studied the corrosion behavior of low-alloy steel in anoxic simulated groundwater using the electrochemical impedance method at 90 °C and the results showed that the corrosion rate decreased with the increase in reaction time. The research on the candidate canister materials for a deep geological repository mainly focused on iron-based materials, such as low-carbon steel, low-alloy steel and nodular cast iron [18,19,20,21,22], while as the basic of iron-based materials, pure iron has rarely been studied regarding its corrosion behavior in a deep geological disposal repository.

Pure iron can be oxidized by water ferric iron via ferrous iron even in an anoxic solution without strong oxidants, i.e., O_2_, H_2_O_2_ and so on [23]. Consistent with the anoxic corrosion of pure iron, the solution pH and corrosion products increase while Eh decreases. In addition, co-existing ions in the solution have different effects on the corrosion of pure iron under anoxic conditions. To be specific, cations like As(V), Se(VI) can be reduced by pure iron nanoparticles [24], while anions like nitrate and sulfate may lower the reduction rate of Se(VI) [25]. Therefore, it is important to investigate the effects of other anions, like Cl^−^, HCO_3_^−^, CO_3_^2−^ and SiO_3_^2−^, which are typical in underground water, on the anoxic corrosion of pure iron.

In this paper, the corrosion behavior of pure iron in anoxic simulated groundwater was studied. Specifically, the anoxic simulated groundwater (SG) solution was simulated by using 10 mM NaCl and 2 mM NaHCO_3_ [1], with different concentrations of CaCl_2_, Na_2_CO_3_ and Na_2_SiO_3_, respectively. The corrosion behavior of pure iron coupons in these simulated solutions was studied via an immersion test, electrochemical test and surface characterization. The effect of temperature was also evaluated by varying the temperature from 25 to 60 °C. Based on the results, a corrosion mechanism of pure iron affected by different ions and temperatures in anoxic simulated groundwater is proposed.

## 2. Experimental

### 2.1. Materials

The chemical composition of the commercial iron plate (> 99.99%) is shown in Table 1. For the immersion experiment, the iron plate was cut into specimens with dimension of 20 mm × 10 mm × 2 mm. Each sample was mechanically grounded to 2000 grit by silicon carbide papers, washed and dried with ethanol and then stored in a desiccator. For the electrochemical experiment, the iron plate was cut into specimens with dimension of 10 mm× 10 mm× 2 mm to make electrodes. The prepared working electrodes were grounded to 2000 grit and polished to a mirror surface, after which they were washed and dried with ethanol and then stored in a desiccator.

### 2.2. Simulated Groundwater Solution

The simulated groundwater includes the major component of an SG solution (10 mM NaCl + 2 mM NaHCO_3_) and different ions (Ca^2+^, CO_3_^2−^, SiO_3_^2−^). The concentrations of ions were set as 0, 1, 5, 10 and 20 mM, respectively, by adding CaCl_2_, Na_2_CO_3_ and Na_2_SiO_3_ solution. The details of the chemical composition of the simulated groundwater for the different groups are shown in Table 2.

### 2.3. Electrochemical Measurement

In order to figure out the corrosion behavior of pure iron in the anoxic simulated groundwater, the measurements of the potentiodynamic polarization curve and electrochemical impedance spectroscopy (EIS) of the iron samples were carried out using a Zahner electrochemical workstation, Germany, with varied ions concentrations (see Table 2) and temperatures (25, 40 and 60 °C). An electrochemical cell of three electrodes (the iron sample acted as the working electrode, a saturated Hg/HgCl electrode worked as the reference electrode and a platinum foil served as the counter electrode) was set up, followed by purging the respective solution with argon gas (containing 0.3% CO_2_) for 20 min to obtain the H_2_- and O_2_-free solution. Then, the open-circuit potential was tested for 20 min and when the potential was stable, EIS was tested with a direct current potential of 0 V relative to the open-circuit, alternate current (AC) amplitude of 10 mV, initial frequency of 10,000 Hz and termination frequency of 0.01 Hz. Finally, the potentiodynamic polarization curve was tested with a potential range of −0.3~+0.8/1.2 V relative to the open-circuit potential. The potential sweep rate is 0.5 mV/s.

### 2.4. Immersion Test

Immersion tests of iron samples were carried out in 20 mM SG and SG + CaCl_2_/Na_2_CO_3_/Na_2_SiO_3_ solution at 25, 40 and 60 °C for 4 weeks, which were replaced each week. Parallel experiments were performed and three samples were taken out after 4 weeks. Two parallel samples used for the determination of the corrosion rate were firstly washed by a rust removal solution (500 mL HCl, 500 mL deionized water and 10 g hexamethylenetetramine), followed by drying and weighing.

The rest of the sample was directly stored in a desiccator for the characterization of the corrosion product and passivation film. The solutions (pH 8.2) were maintained anoxic by purging Ar gas (containing 0.3% CO_2_) prior to the experiment and during the replacement of the solutions.

### 2.5. Surface Characterization

Scanning electron microscopy with energy dispersive spectroscopy (SEM/EDS) was conducted to analyze the surface morphologies and elemental compositions of the iron surfaces after the immersion tests, by using a FEI Quanta FEG 250 SEM equipped with an Oxford Inca X-act 2000 EDS system (FEI, Hillsboro, OR, USA). An accelerating voltage of 15 kV and a working distance of 10 mm were used to analyze secondary electron images.

Confocal Raman microscopy (CRM, Renishaw, London, UK) measurements were carried out to analyze the formation of the corrosion products after the immersion tests using a Renishaw inVia Qontor CRM system equipped with a laser source with the wavelength of 532 nm. The scan range was 0–1200 cm^−1^ with a spectral resolution of 1 cm^−1^.

X-ray photoelectron spectroscopy (XPS, Kratos, Manchester, UK) measurements were performed to characterize the formation of the passivation film after the immersion tests by using a Kratos AXIS Ultra DLD spectrometer, with a monochromated Al K-α X-ray source (hv = 1486.69 eV) at the power of 150 W. The working voltage was set as 15 kV and the transmission current was set as 10 mA. The chemical state assessment was achieved by curve-fitting the spectra using the XPSpeak software (XPSpeak4.1, Hong Kong, China).

## 3. Result and Discussion

### 3.1. Potentiodynamic Polarization Curves

**Figure 1:**
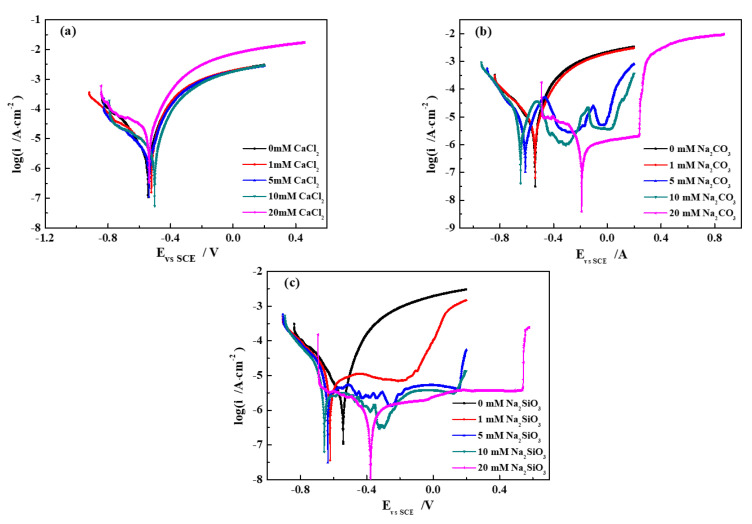
Polarization curves of pure iron in different simulated groundwaters at 40 °C. (**a**) SG + CaCl_2_; (**b**) SG + Na_2_CO_3_; (**c**) SG + Na_2_SiO_3_.

**Figure 2:**
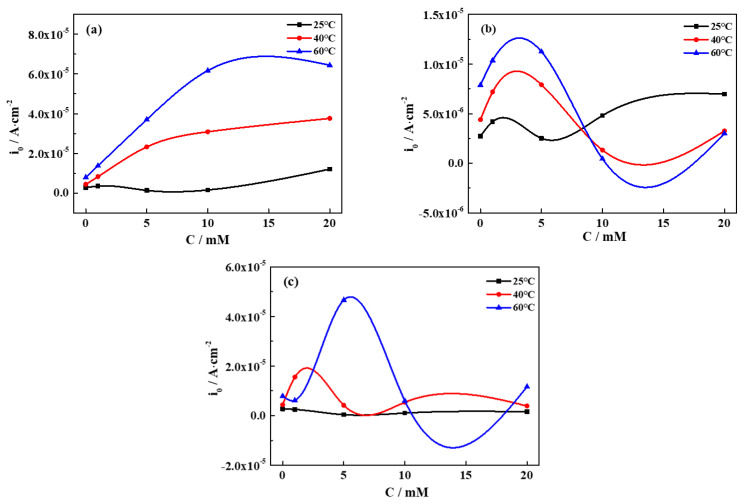
The variation in the corrosion current density with concentration/temperature in different simulated groundwaters. (**a**) SG + CaCl_2_; (**b**) SG + Na_2_CO_3_; (**c**) SG + Na_2_SiO_3_.

The electrochemical corrosion behavior of pure iron in different groundwater solutions and at different temperatures was investigated by potentiodynamic polarization measurements. The polarization curves measured at 40 °C are shown in Figure 1, indicating distinguishing characteristics in different simulated groundwater solutions. By means of the Tafel extrapolation method, electrochemical parameters obtained from the fitted curves are presented in Table 3, showing the corrosion current density and corrosion potential. The variation in the corrosion current density with varying concentrations/temperatures is displayed in Figure 2.

In the SG + CaCl_2_ solution (Figure 1a), the curves are very similar, characterized as active dissolution control in both the anodic and cathodic areas. The anodic polarization curves appear in the active dissolution region, but are without occurrence in the passivation region [26,27]. Both the SG and CaCl_2_ solutions contain active Cl^−^, which easily destroys the protective oxide film formed on the iron surface and triggers the dissolution of the iron, further accelerating the electrochemical corrosion activity.

Seen from Table 3 and Figure 2a, it is evident that the corrosion current density demonstrates a rising trend with increased concentrations of CaCl_2_ and temperature, suggesting a higher corrosion activity of iron. The increase in concentration and temperature accelerates the activity of Cl^−^ and promotes the dissolution of the corrosion products, leading to the increase in the corrosion rate [21]. The corrosion rate of iron in the SG + CaCl_2_ solution is higher than that in other environments, indicating Cl^−^ plays dominant roles in the electrochemical processes.

In the SG + Na_2_CO_3_ solution (Figure 1b), the polarization curves in the 0 and 1 mM Na_2_CO_3_ solutions are very similar to Figure 1a, indicating Cl^−^ plays a major role in the kinetic control of active dissolution. However, with increased concentrations of Na_2_CO_3_ (5 and 10 mM), the polarization curves display an obvious inflexion after −0.55 V and then appear as fluctuating transition and passivation regions. It suggests that a discontinuous passivation film gradually forms on the iron surface. When the concentration of Na_2_CO_3_ reaches 20 mM, the corrosion potential positively shifts to −0.2 V and the obviously different curves show very stable passivation and over passivation regions, but without a transition passivation region. It is evident that the high concentration of CO_3_^2−^ is related to the stable passivation region in the anodic polarization curve, indicating the formation of a continuous passivation film on the iron surface.

Seen from Table 3 and Figure 2b, with increased concentrations of Na_2_CO_3_, the overall trend of the corrosion current density firstly rises and then falls, and gradually becomes stable. The effects of active Cl^−^ competes with the formation of the passivation film, so the fluctuated corrosion rates are dependent on the synergetic effect. However, the effects of temperature on the corrosion current density are not consistent, which is dependent on the concentration of the solution. In the low-concentration solution, the corrosion current density increases with the increased temperature, which is in relation to the enhanced activity of Cl^−^. The discontinuous passivation film formed in the low-concentration solution is easily destroyed by Cl^−^, resulting in an increased corrosion rate of iron. However, a high concentration of CO_3_^2−^ promotes the formation of the passive film, further preventing the penetration of Cl^−^. The decreased corrosion current density attributes to the barrier effects of the passivation film, resulting in a lower corrosion rate with the increased temperature.

In the SG + Na_2_SiO_3_ solution (Figure 1c), the characteristics of the polarization curves are very similar to the Na_2_CO_3_ solution. With increased concentrations of Na_2_SiO_3_, the passivation region becomes more and more stable. When the concentration of Na_2_SiO_3_ reaches 20 mM, the wide potential range of the passivation region infers the formation of a denser and more uniform passive film than that formed in the Na_2_CO_3_ solution.

Seen from Table 3 and Figure 2c, similar to the Na_2_CO_3_ solution, the variation in the corrosion current density is fluctuating and not stable with the increased concentrations of Na_2_SiO_3_, indicating the competition of synergetic effects between the active Cl^−^ and the passivation film. The effects of temperature on the corrosion current density are also dependent on the concentration. On the one hand, a high temperature promotes SiO_3_^2−^ to form a dense and uniform passivation film. On the other hand, a high temperature accelerates Cl^−^ to destroy the passivation film.

### 3.2. Electrochemical Impedance Spectroscopy

**Figure 3:**
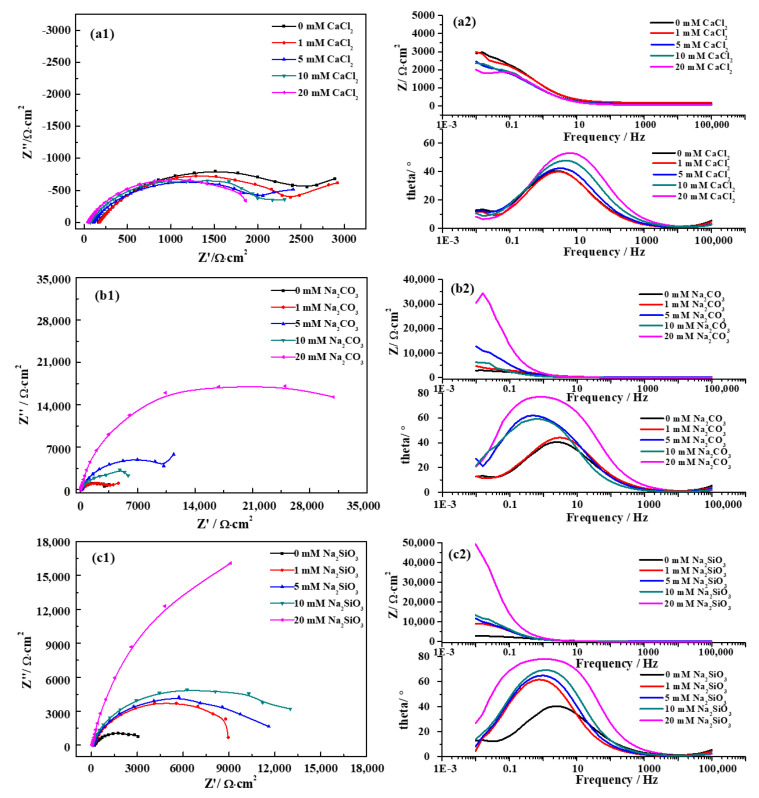
EIS Nyquist (1) and Bode (2) spectra of pure iron in different simulated groundwaters at 40 °C. (**a**) SG + CaCl_2_; (**b**) SG + Na_2_CO_3_; (**c**) SG + Na_2_SiO_3_.

**Figure 4:**
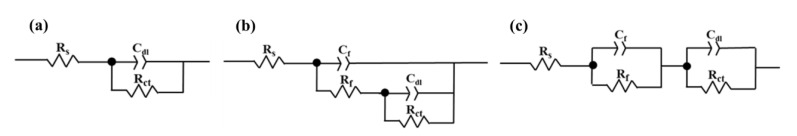
Equivalent electric circuits used to extract parameters from the EIS spectra. (**a**) SG + CaCl_2_; (**b**) SG + Na_2_CO_3_; (**c**) SG + Na_2_SiO_3_. (R_s_: electrolyte resistance; R_ct_: charge-transfer resistance; R_f_: film resistance; C_dl_: double layer capacitance; C_f_: film capacitance).

**Figure 5:**
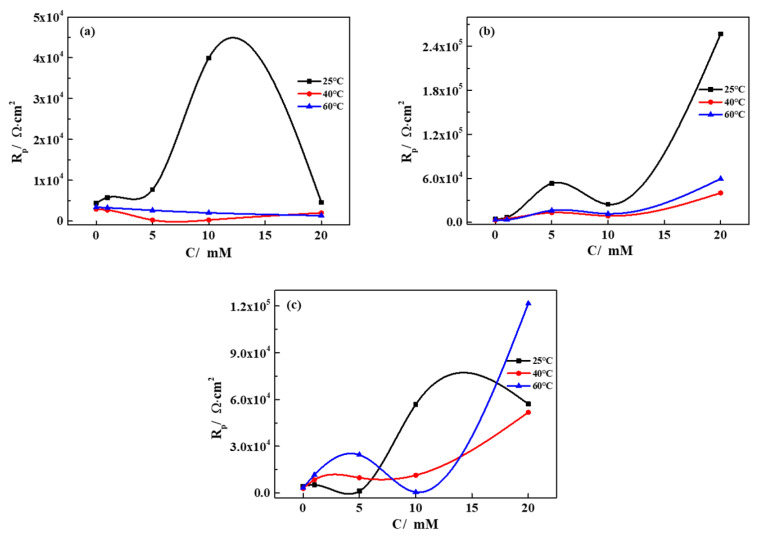
The variation in the polarization resistance of iron with concentration/temperature in different simulated groundwaters. (**a**) SG + CaCl_2_; (**b**) SG + Na_2_CO_3_; (**c**) SG + Na_2_SiO_3_.

Electrochemical impedance measurements were performed to characterize the barrier effect of the oxide film formed on the pure iron in different groundwater solutions. The EIS spectra measured at 40 °C are shown in Figure 3, displaying different characteristics in different groundwater solutions. Electrochemical parameters obtained from the fitted EIS spectra are presented in Table 4, based on a different equivalent circuit (Figure 4). In order to illuminate the effect of different ions and temperature on the corrosion resistance of iron in simulated groundwater, the variation in the polarization resistance with varying concentrations/temperatures are displayed in Figure 5.

In the SG + CaCl_2_ solution (Figure 3a), the Nyquist spectra show only one capacitive loop and the diameter of the loop decreases with the increased concentration of CaCl_2_. From the Bode plot, it can be seen that the major process has a capacitive slope below 10 Hz. With the increased concentration, the resistance of pure iron at low frequency decreased from about 3000 to 2000 Ω·cm^2^. A time constant is evident at low frequency, probably assigned to a charge transfer process [28]. The equivalent circuit used for modeling the Nyquist plots consisted of electrolyte resistance (R_s_), charge-transfer resistance (R_ct_) and double layer capacitance (C_dl_) (Figure 5a). The polarization resistance (R_p_) equals to R_ct_.

Seen from Table 4 and Figure 4a, the polarization resistance is basically conserved with the increased concentration of CaCl_2_ (10 mM at 25 °C is the only exception), indicating the corrosion resistance of iron is independent from the concentration. Similar to the polarization results, the effects of the formation of the passivation film compete with the active Cl^−^, which is not beneficial to enhance corrosion resistance. However, the effects of the increased temperature on polarization resistance were generally decreasing, indicating that the high temperature accelerated the activity of Cl^−^ and promoted the dissolution of the corrosion products, resulting in a lower corrosion resistance. The corrosion resistance of iron in the SG + CaCl_2_ solution was much lower than that in other environments, indicating Cl^−^ plays dominant roles in the electrochemical processes.

In the SG + Na_2_CO_3_ solution (Figure 3b), a capacitive loop appears in the Nyquist spectra. Different from the CaCl_2_ solution, the diameter of the loop increases with the increased concentration of Na_2_CO_3_. The Bode plot indicates that the major process has a capacitive slope below 1 Hz. With the increased concentration, the resistance of pure iron at low frequency increased from about 3000 to 34,000 Ω·cm^2^. A time constant is also evident at low frequency, probably assigned to a charge transfer process. However, with the increased concentration, the characteristics of the peaks of the phase angle changed from symmetry to asymmetry, suggesting the possible existence of two overlapped peaks [29,30]. Hence, two time constants were required for a proper fitting, indicating two electrochemical reactions occurred on the iron surface. Based on the results from the polarization curves, the high concentration of CO_3_^2−^ promoted the formation of the passive film, so Cl^−^ plays an important role in the dissolution of both the passivation film and iron matrix. The equivalent circuit used for modeling the Nyquist plots consists of electrolyte resistance (R_s_), film resistance for passivation film formed on the iron surface (R_f_), film capacitance (C_f_), charge-transfer resistance (R_ct_) and double layer capacitance (C_dl_) (Figure 5b). The polarization resistance (R_p_) is calculated by adding R_f_ and R_ct_.

Seen from Table 4 and Figure 5b, it is indicated that raising the concentration of Na_2_CO_3_ increases both R_f_ and R_p_ of pure iron. It is obvious that CO_3_^2−^ promotes the formation of the passive film, further preventing the penetration of Cl^−^. The passivation film becomes uniform and dense with the increased concentration, demonstrating higher barrier effects on the surface. Even though the corrosion rate is fluctuant, shown in the polarization curves, the corrosion resistance of iron largely enhances with the increased concentration. However, the polarization resistance decreased with the increased temperature, which is related to the reduced barrier effects of the passivation film. The high temperature easily accelerates the activity of Cl^−^, leading to the dissolution of the passivation film, further lowering the corrosion resistance.

In the SG + Na_2_SiO_3_ solution (Figure 3c), the characteristics of the EIS spectra are similar to the Na_2_CO_3_ solution. With the increased concentration, the resistance of pure iron at low frequency increased from about 3000 to 50,000 Ω·cm^2^, which is even higher than the resistance in the Na_2_CO_3_ solution. Due to the asymmetry peaks of the phase angles, two time constants were also required for a proper fitting. The equivalent circuit used for modeling the Nyquist plots consists of the same components used for the Na_2_CO_3_ solution, but two series of networks were used to address the effect of a dense and uniform passivation film.

Seen from Table 4 and Figure 5c, the variation in the polarization resistance shows a fluctuating but increasing trend in the SG + Na_2_SiO_3_ solution (20 mM at 25 °C is the only exception). Similar to the Na_2_CO_3_ solution, with the increased concentration, an increased R_f_ evidently indicates the formation of a uniform and dense passivation film, which supplies a high barrier effect on the surface. Even though the corrosion rate is fluctuant with the concentration, shown in the polarization curves, the corrosion resistance is enhanced, showing an even larger value compared with Figure 5b. Hence, SiO_3_^2−^ promotes the formation of a denser and more uniform passivation film compared with CO_3_^2−^. However, the effects of temperature on the corrosion current density are also dependent on the concentration, similar to the results from the polarization curves. The formation of the passivation film competes with the active Cl^−^, so the corrosion resistance is dependent on the synergetic effect. The synergetic effects of CO_3_^2−^/SiO_3_^2−^ and Cl^−^ contribute to a dynamic process in the electrochemical reactions, leading to repeated passivation–dissolution processes for the formation of the passivation film.

### 3.3. Corrosion Rate Obtained from Immersion Tests

**Figure 6:**
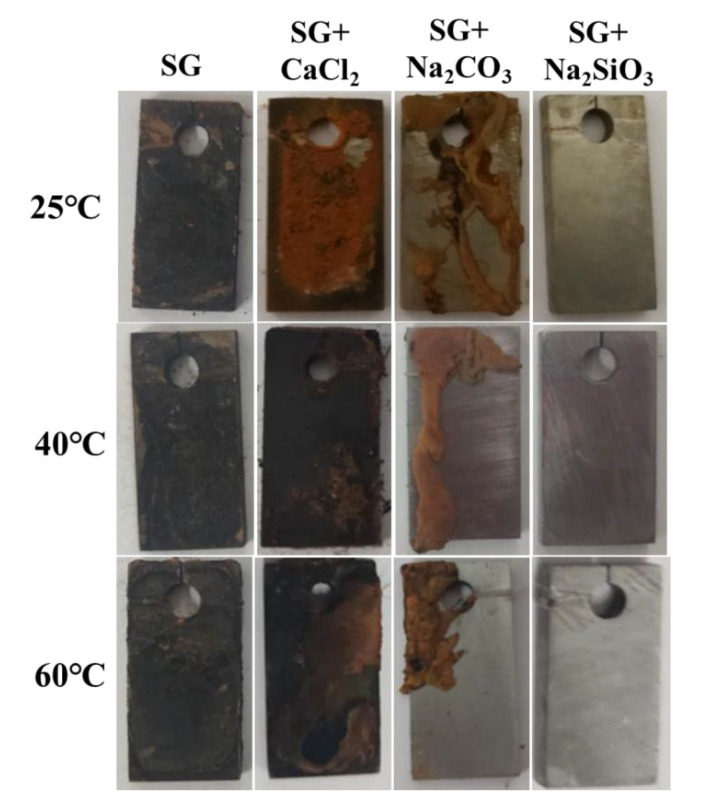
Visual appearance of iron after four weeks immersion in 20 mM SG and SG + CaCl_2_/Na_2_CO_3_/Na_2_SiO_3_ solutions.

In order to investigate the corrosion rate of iron samples, immersion tests were carried out in simulated groundwater with the addition of 20 mM ions. After four weeks of exposure, the corrosion products were removed by a mixed solution of 500 mL HCl, 500 mL deionized H_2_O and 10 g C_6_H_12_N_4_. The visual appearances of the corroded samples’ surfaces are shown in Figure 6. The surfaces after immersion in the SG solution and the SG + CaCl_2_ solution are completely covered with a thick layer of corrosion products. In the SG + Na_2_CO_3_ solution, the surfaces are partially covered with corrosion products, mainly located at the side and edge of the samples. Besides corrosion products, other parts of the surfaces are not corroded. However, the visual appearances of the surfaces after immersion in the SG + Na_2_CO_3_ solution are quite different, showing barely an iron matrix covered by a thin layer of passivation film.

The corrosion rate of the iron samples after the immersion test was calculated by the weight loss method. The calculation formula of the corrosion rate is as follows [31]:

(1) $$\mathrm{CR}\;\left(\mathrm{mm}/y\right)=\frac{87600\times \left(M-M_{1}\right)}{S\times T\times \rho }$$

where CR represents the corrosion rate (mm/y, corrosion depth per year), M represents the weight of the sample before immersion (g), M_1_ represents the weight of the sample after four weeks of immersion (g), S represents the total surface area of the sample (cm^2^), T represents the immersion period (H) and d represents the density of the sample (g/m^3^). The results are expressed as an average corrosion rate, which was calculated by two samples.

**Figure 7:**
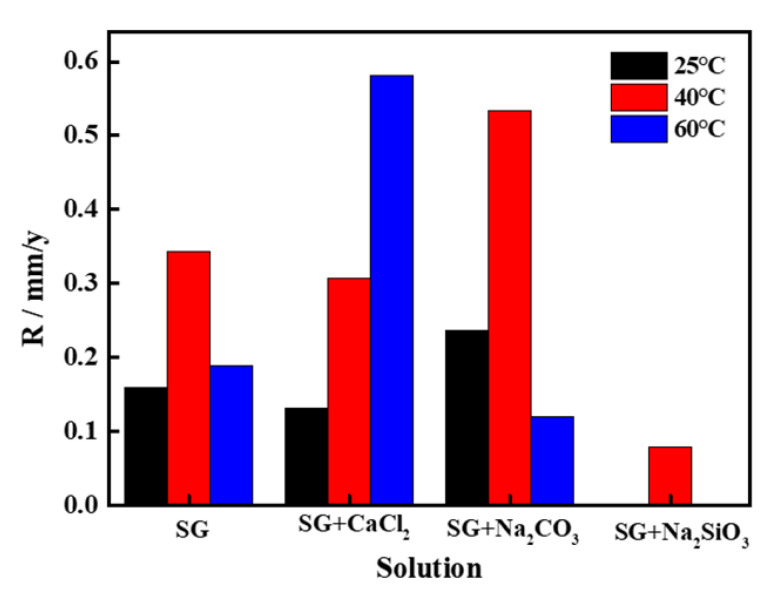
Weight loss corrosion rates of iron after immersion tests in different simulated groundwaters.

The weight loss corrosion rates of the pure iron in different simulated groundwaters are compared in Figure 7. It seems that the general corrosion rates of iron in the SG/SG + CaCl_2_/SG + Na_2_CO_3_ solution are much higher than that in the SG + Na_2_SiO_3_ solution, consistent with the electrochemical results. In the SG + CaCl_2_ solution, the corrosion rates increased with the increased temperature, showing an accelerated corrosion activity of iron. In the SG + Na_2_CO_3_/Na_2_SiO_3_ solution, the corrosion rates are also fluctuant with the increased temperature. In agreement with the electrochemical results, it is indicated that Cl^−^ competes with the passivation film, contributing synergetic effects to the corrosion rate. The corrosion rates of iron with the addition of Na_2_SiO_3_ at 25 and 60 °C are approximately zero, which indicates that the barrier effects of the passivation film largely protect the iron matrix.

### 3.4. Characterization of Corrosion Products and Passivation Film

In order to analyze the morphology and composition of the corrosion products and passivation film, SEM/EDS, CRM and XPS measurements were performed on the iron surfaces after the immersion tests.

**Figure 8:**
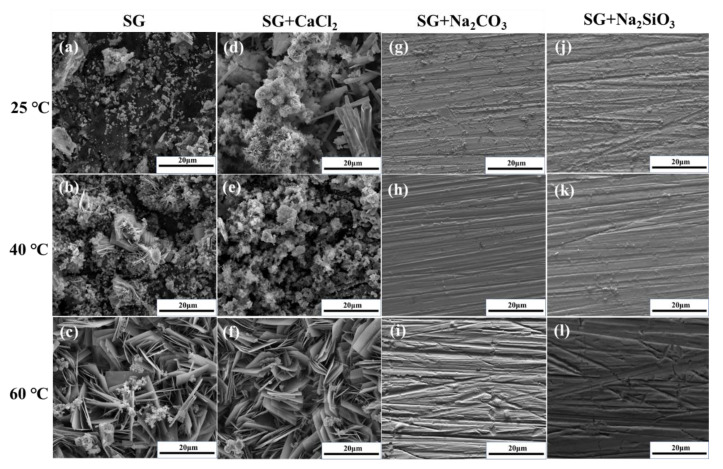
Surface morphology of iron after 4 weeks immersion in 20 mM SG and SG + CaCl_2_/Na_2_CO_3_/Na_2_SiO_3_ solutions (**a**–**l**).

Seen from Figure 8a–f, the characteristics of the corrosion products change with different ions and temperatures in the SG and SG + CaCl_2_ solutions. In the SG solution, granular corrosion products are partially distributed on the iron surface at 25 °C. Corrosion products gradually accumulate together, and it was found that flower-like clusters appear with the increased temperature. In the SG + CaCl_2_ solution, a loose layer of corrosion products was found, consisting of flower-like and rod-like clusters. With the increased temperature, the corrosion products clusters become larger and larger, showing a distinguished lamellar structure.

The morphology of the passivation film formed in the SG + Na_2_CO_3_/Na_2_SiO_3_ solution is displayed in Figure 8g–l. Different from the formation of a large number of corrosion products in the SG and SG + CaCl_2_ solutions, only a small amount of corrosion products are partially distributed on the iron surface. With the increased temperature, the amount of corrosion products further decreases and scratches on the iron surface could be observed, indicating the substrate is protected by a thin film of the passivation layer.

When comparing the distribution of the corrosion products formed with the different ions, it can be seen that adding CO_3_^2−^ and SiO_3_^2−^ significantly reduces the amount of corrosion products, showing the characteristics of the passivation film. When comparing the different temperatures in the SG and SG + CaCl_2_ solutions, it can be seen that the corrosion products clusters significantly accumulated and grew in size, indicating that a high temperature will promote the electrochemical reactions and accelerate the corrosion behavior of iron. However, with the increased temperature in the SG + Na_2_CO_3_/Na_2_SiO_3_ solution, the increased barrier effect of the passivation film protects the substrate, further reducing the amount of corrosion products.

**Figure 9:**
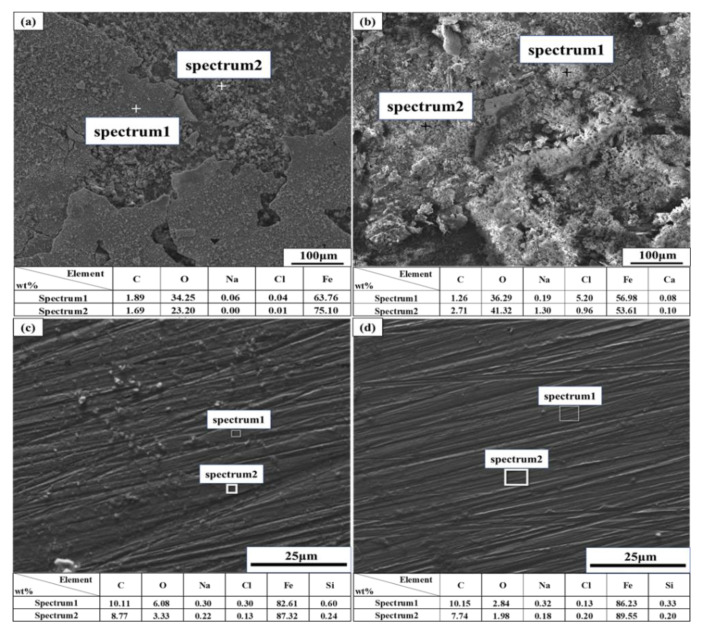
EDS analysis of iron after 4 weeks immersion in different simulated groundwaters. (**a**) SG solution. (**b**) SG + 20 mM CaCl_2_; (**c**) SG + 20 mM Na_2_CO_3_; (**d**) SG + 20 mM Na_2_SiO_3_.

The corresponding EDS analyses of the corrosion products are displayed in Figure 9. The corrosion products formed in the SG and SG + CaCl_2_ solutions are composed of Fe and O, inferring different types of iron oxide. Ca and C are also detected on the iron surface after immersion in the SG + CaCl_2_ solution. Although Ca^2+^ may react with HCO_3_^−^ to form calcium-rich products, it seemed the products failed to protect the iron surface due to the strong destroying ability of Cl^−^. In the SG + Na_2_CO_3_/Na_2_SiO_3_ solution, the main components of the passivation film are composed of Fe, O, C and Si. Carbonate-rich and silicate-rich passivation films possibly form on the iron surface during electrochemical reactions, which prevent the penetration of Cl^−^, resulting in a protective effect to lower the corrosion rate.

**Figure 10:**
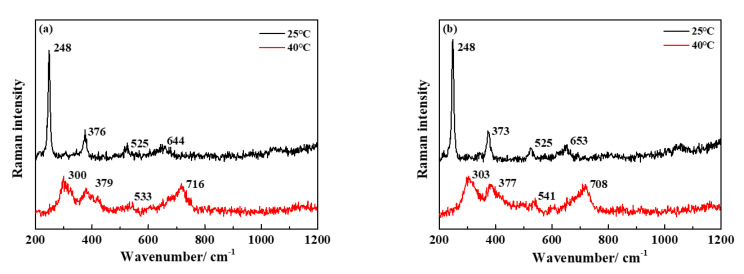
Raman spectra obtained on iron surfaces after 4 weeks immersion in the SG (**a**) and SG + 20 mM CaCl_2_ (**b**) solutions.

CRM measurements after the immersion tests were conducted to identify the distribution of the corrosion products on the iron surfaces in the SG and SG + CaCl_2_ solutions. The Raman spectra in Figure 10 reveal that the formations of corrosion products are very similar without or with the addition of CaCl_2_. In Figure 10a, it is obvious that the corrosion products mainly consist of lepidocrocite (γ-FeOOH; associated with the bands at 248, 376, 525 and 644 cm^−1^) at 25 °C. When the temperature increases to 40 and 60 °C, besides the formation of lepidocrocite, the corrosion products are mainly composed of goethite (α-FeOOH; associated with the bands at 300, 379 and 533 cm^−1^) and maghemite (γ-Fe_2_O_3_; associated with band at 716 cm^−1^) [32,33,34]. In the SG + CaCl_2_ solutions, Figure 10b demonstrates a similar trend of corrosion products formation, which is mainly composed of γ-FeOOH, α-FeOOH and γ-Fe_2_O_3_. However, the passivation film formed in the SG + Na_2_CO_3_/Na_2_SiO_3_ solution is too thin to be detected by the CRM measurements.

**Figure 11:**
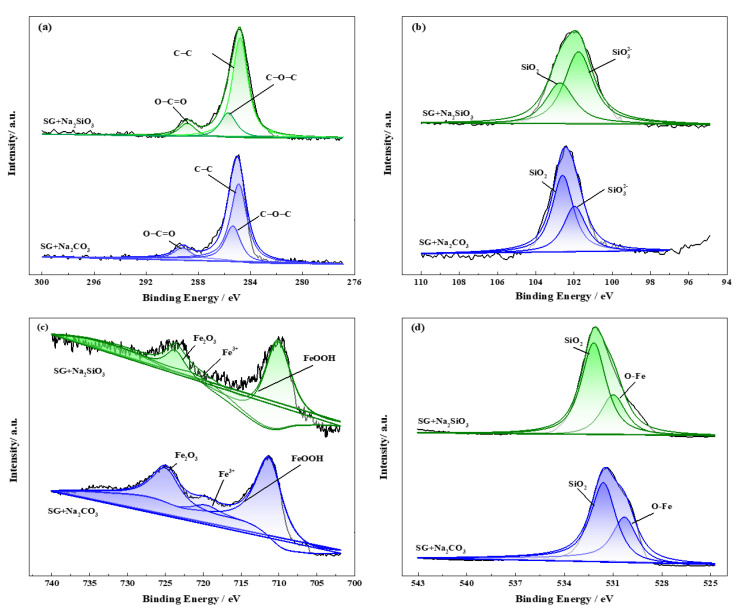
XPS spectra obtained on iron surfaces after 4 weeks immersion in the SG + 20 mM Na_2_CO_3_/Na_2_SiO_3_ solution. (**a**) C 1s; (**b**) Si 2p; (**c**) Fe 2p; (**d**) O 1s.

High-resolution XPS spectra were performed to achieve detailed information about the passivation films formed in the SG + Na_2_CO_3_/Na_2_SiO_3_ solution. From the patterns shown in Figure 11a, the C 1s core levels were deconvoluted into three different components of C-C (284.8 eV), C-O-C (285.7 eV) and O-C=O (289.1 eV) bonds, indicating the possible existence of CO_3_^2−^ and CO_2_ [35]. The Si 2p core levels, shown in Figure 11b, were deconvoluted into two different components. The component with a binding energy around 102.6 eV is reported for silicon bonded to oxygen in the silica compound (SiO_2_), where the component with a lower binding energy (101.9 eV) is assigned to the silicate-based species [36,37]. The formation of SiO_2_ is attributed to the oxidation of Si in the iron substrate. The silicate content is significantly higher for the passivation film formed in the SG + Na_2_CO_3_ solution, whereas SiO_2_ predominates for the film formed in the SG + Na_2_SiO_3_ solution. From Figure 11c, the complex Fe2p core level structure is composed of three components. Two pronounced satellite peaks with a binding energy around 711.4 and 725.0 eV correspond to nonstoichiometric FeOOH and Fe_2_O_3_, respectively, which is possibly connected to the small amount of corrosion products (Figure 8g,j). Another weak satellite peak at 720.0 eV is assigned to the Fe^3+^ species [36,38], indicating the possible existence of ferric carbonate and silicate. The O 1s spectra (Figure 11d) can be deconvoluted into two components of SiO_2_ (531.6 eV) and Fe-O bonds (530.3 eV) [38]. Based on the XPS information, it is evident that the passivation film formed in SG + Na_2_CO_3_/Na_2_SiO_3_ is mainly composed of SiO_2_, ferric carbonate and silicate, where ferric silicate is dominant with the addition of SiO_3_^2−^.

### 3.5. Mechanism of Corrosion Behavior of Iron in Simulated Groundwater

**Figure 12:**
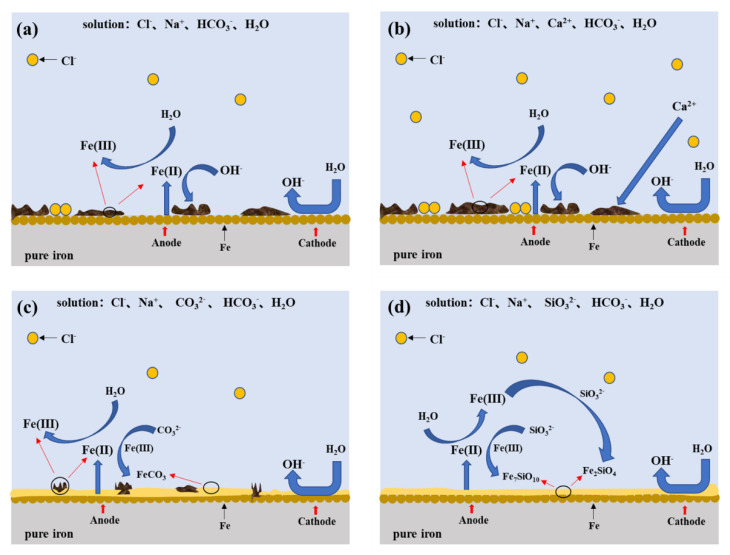
The mechanistic scheme of corrosion behavior of iron in simulated groundwaters. (**a**) SG; (**b**) SG + CaCl_2_; (**c**) SG + Na_2_CO_3_; (**d**) SG + Na_2_SiO_3_.

The mechanism of the corrosion behavior of iron in simulated groundwater is elucidated in Figure 12. The effects of different ions and temperatures on the corrosion behavior are discussed based on the electrochemical results, weight loss corrosion rates and surface characterization.

Seen from Figure 12a,b, both the SG and SG + CaCl_2_ solutions contain active Cl^−^, which easily destroys the protective oxide film formed on the iron surface and triggers the dissolution of iron, further accelerating the electrochemical corrosion processes. The corrosion products formed on the iron surface mainly consist of γ-FeOOH, α-FeOOH and γ-Fe_2_O_3_. The increased concentration and temperature accelerate the activity of Cl^−^ and promote the dissolution of the corrosion products, leading to the increase in the corrosion rate. Although Ca^2+^ may react with OH^−^ to form calcium-rich products, it seems the products are easily destroyed by Cl^−^. Hence, the effects of Ca^2+^ on the corrosion behavior of iron is negligible, however, the enhanced effects of chloride ions with temperature lead to more severe corrosion.

In summary, the anodic and cathodic reactions during the corrosion process of pure iron in the anoxic SG and SG + CaCl_2_ solutions occurred as follows [23,24,39]:

Anodic reaction:

(2) $$Fe⟶Fe^{2+}+2e^{-}$$

Cathodic reaction:

(3) $$2H_{2}O+2e^{-}⟶2OH^{-}+H_{2}$$

Overall reactions:

(4) $$Fe+2H_{2}O⟶Fe{\left(OH\right)}_{2}+H_{2}$$

(5) $$2Fe+3H_{2}O⟶Fe_{2}O_{3}+3H_{2}$$

(6) $$2Fe{\left(OH\right)}_{2}+2H_{2}O⟶2Fe{\left(OH\right)}_{3}+H_{2}$$

(7) $$Fe{\left(OH\right)}_{3}⟶FeOOH+H_{2}O$$

(8) $$Ca^{2+}+2OH^{-}⟶Ca{\left(OH\right)}_{2}$$

In the SG + Na_2_CO_3_/Na_2_SiO_3_ solution, shown in Figure 12c,d, additional CO_3_^2−^ and SiO_3_^2−^ play important roles in the formation of the passivation film, reducing the formation of corrosion products on the iron surface with the increased concentration. However, the effects of temperature on the corrosion rates are not consistent. Generally, in the low-concentration solution, Cl^−^ plays important roles in enhancing the corrosion activity, so the corrosion rates increase with the increased temperature. A uniform passivation film gradually forms with the increased concentration of CO_3_^2−^ and SiO_3_^2−^, composed of protective SiO_2_, ferric carbonate and silicate. In the high-concentration solution, on one hand, the passivation film plays an important role in enhancing the barrier effects. On the other hand, Cl^−^ accelerates the dissolution processes with the increased temperature. The synergistic effects lead to repeated passivation–dissolution processes in the formation of the passivation film, leading to a fluctuant corrosion rate varying with the temperature.

In summary, the anodic and cathodic reactions during the corrosion process of pure iron in the anoxic SG + Na_2_CO_3_/Na_2_SiO_3_ solution occurred as follows:

Anodic reaction [23,40]:

(9) $$Fe⟶Fe^{2+}+2e^{-}$$

Cathodic reaction:

(10) $$2H_{2}O+2e^{-}⟶2OH^{-}+H_{2}$$

Overall reactions:

(11) $$Fe^{2+}+CO_{3}^{2-}⟶FeCO_{3}$$

(12) $$2Fe^{2+}+SiO_{3}^{2-}+2OH^{-}⟶Fe_{2}SiO_{4}+H_{2}O$$

(13) $$5Fe^{2+}+2Fe^{3+}+SiO_{3}^{2-}+14OH^{-}⟶Fe_{7}SiO_{10}+7H_{2}O$$

Hence, the effects of $CO_{3}^{2-}$ and $SiO_{3}^{2-}$ are beneficial to form a passivation film in anoxic simulated groundwater. In the Na_2_SiO_3_ solution, the formation of ferric silicate predominates, resulting in a more uniform and denser passivation film compared with that formed in the Na_2_CO_3_ solution. The penetration of Cl^−^ is largely prevented, improving the protective effects of the iron surfaces.

## 4. Conclusions

The effects of Ca^2+^, CO_3_^2−^ and SiO_3_^2−^ on the corrosion behavior of pure iron in SG solutions were investigated by varying the concentration of added ions and reaction temperatures. The conclusions are as follows:

1. The corrosion rate of pure iron in the SG + CaCl_2_ solution increases with the increasing concentration and temperature. The effects of Ca^2−^ on the corrosion behavior of iron is negligible, however, Cl^−^ plays important roles in the corrosion processes. The increased concentration and temperature accelerate the activity of Cl^−^ and promote the dissolution of corrosion products, leading to more severe corrosion behavior. The formation of the corrosion products is mainly consisted of γ-FeOOH, α-FeOOH and γ-Fe_2_O_3_ in the SG and SG + CaCl_2_ solutions;
2. The corrosion resistance of iron is largely improved by adding CO_3_^2−^ and SiO_3_^2−^ in the SG + Na_2_CO_3_/Na_2_SiO_3_ solution. A uniform passivation film gradually forms with the increased concentration, playing important roles in increasing the barrier effects of the iron surface and decreasing the formation of corrosion products. The passivation film is mainly composed of SiO_2_, ferric carbonate and silicate;
3. The effects of temperature on the corrosion behavior of iron in the SG + Na_2_CO_3_/Na_2_SiO_3_ solution are not consistent. Cl^−^ is dominant in enhancing the corrosion activity in the low-concentration solution, so the corrosion rates increase with the increased temperature. In the high-concentration solution, the synergistic effects of CO_3_^2−^/SiO_3_^2−^ and Cl^−^ contribute to the synergistic effects in the formation of the passivation film, leading to the cycles of passivation–dissolution processes. Hence, the corrosion rates are fluctuant with the varying temperature;
4. The effects of CO_3_^2−^ and SiO_3_^2−^ are beneficial to iron in simulated groundwater. The formation of ferric silicate is dominant with the addition of SiO_3_^2−^, resulting in a more uniform and denser passivation film than the film formed with the addition of CO_3_^2−^. The penetration of Cl^−^ is effectively prevented and the corrosion resistance of iron is largely improved.

## Figures and Tables

**Table 1:** Chemical composition of pure iron.

Material	Composition (wt.%)					
	C	S	P	Si	Mn	Fe
Pure iron	0.003	0.002	0.002	0.002	0.001	balance

**Table 2:** Chemical composition of simulated groundwater.

Solution	Chemicals Concentration (mM)				
	NaCl	NaHCO_3_	CaCl_2_	Na_2_CO_3_	Na_2_SiO_3_
SG	10	2	0	0	0
SG + CaCl_2_	10	2	1	0	0
	10	2	5	0	0
	10	2	10	0	0
	10	2	20	0	0
SG + Na_2_CO_3_	10	2	0	1	0
	10	2	0	5	0
	10	2	0	10	0
	10	2	0	20	0
SG + Na_2_SiO_3_	10	2	0	0	1
	10	2	0	0	5
	10	2	0	0	10
	10	2	0	0	20

**Table 3:** Electrochemical parameters obtained from the fitted curves.

40 °C		C/mM	0	1	5	10	20
	i_0_/E_0_						
CaCl_2_	i_0_/A·cm^−2^		4.40 × 10^−6^	8.25 × 10^−6^	2.33 × 10^−5^	3.09 × 10^−5^	3.76 × 10^−5^
	E_0_/V		−0.543	−0.542	−0.542	−0.540	−0.536
Na_2_CO_3_	i_0_/A·cm^−2^		4.40 × 10^−6^	7.19 × 10^−6^	7.91 × 10^−6^	1.33 × 10^−6^	3.27 × 10^−6^
	E_0_/V		−0.543	−0.537	−0.610	−0.642	−0.161
Na_2_SiO_3_	i_0_/A·cm^−2^		4.40 × 10^−6^	1.56 × 10^−5^	4.19 × 10^−6^	5.40 × 10^−6^	3.93 × 10^−6^
	E_0_/V		−0.543	−0.621	−0.634	−0.655	−0.377

**Table 4:** Electrochemical parameters of equivalent circuit of electrochemical impedance spectroscopy (EIS).

Solution	C/mM	R_s_/Ω	R_ct_/Ω	R_f_/Ω	R_p_/Ω	C_r_/S·sec^n^	C_dl_/S·sec^n^
CaCl_2_	0 mM	169.0	2889.0	−	2889.0	−	0.00024
	1 mM	176.8	2653.0	−	2653.0	−	0.000023
	5 mM	108.6	226.4	−	226.4	−	0.00030
	10 mM	72.3	227.1	−	227.1	−	0.00026
	20 mM	44.9	1977.0	−	1977.0	−	0.00022
Na_2_CO_3_	1 mM	174.8	3535.0	1277.0	4812	0.00017	0.0085
	5 mM	115.9	12.7	13,280.0	13,292.7	0.00021	0.000031
	10 mM	68.9	13.5	8780.0	8793.5	0.000052	0.00044
	20 mM	54.9	89.6	40,060.0	40,149.6	0.000063	0.000043
Na_2_SiO_3_	1 mM	168.7	8164.0	275.1	8439.1	0.00020	0.0020
	5 mM	275.1	9494.0	289.1	9783.1	0.00021	0.021
	10 mM	85.1	0.3	11,400.0	11,400.3	0.00023	0.00019
	20 mM	51.6	28.3	51,770.0	51,798.3	0.00016	0.00010
